# Ambient Refractive-Index Measurement with Simultaneous Temperature Monitoring Based on a Dual-Resonance Long-Period Grating Inside a Fiber Loop Mirror Structure

**DOI:** 10.3390/s18072370

**Published:** 2018-07-21

**Authors:** Renata Zawisza, Tinko Eftimov, Predrag Mikulic, Wojtek J. Bock, Leszek R. Jaroszewicz

**Affiliations:** 1 Institute of Applied Physics, Military University of Technology, 2 gen. Witolda Urbanowicza St., 00-908 Warsaw, Poland; jarosz@wat.edu.pl; 2 Photonics Research Center, Université du Québec en Outaouais, 101 Rue St Jean Bosco, Pavillon Lucien Brault, Gatineau, QC J8X 3X7, Canada; Tinko.Eftimov@uqo.ca (T.E.); Predrag.Mikulic@uqo.ca (P.M.); Wojtek.Bock@uqo.ca (W.J.B.)

**Keywords:** optical fiber sensor, dual-resonance long-period grating, fiber loop mirror, temperature control, refractive-index sensor

## Abstract

In this work, we report the experimental results on optimizing the optical structure for ambient refractive index measuring with temperature changes monitoring. The presented optical structure is based on a dual-resonance long-period grating embedded inside a fiber loop mirror, where the long-period grating acts as the head of the refractive-index sensor, whereas the section of polarization maintaining fiber in the loop mirror ensures suitable temperature sensing. The optimization process was comprised of tuning the resonance and interferometric peaks by changing the state of polarization of propagating beams. Experimental results establish that the response of the proposed sensor structure is linear and goes in opposite directions: an increase in the ambient refractive index reduces the signal response, whereas a temperature increase produces an increased response. This enables us to distinguish between the signals from changes in the refractive index and temperature. Due to the filtering properties of the interferometric structure, it is possible to monitor variation in these physical parameters by observing optical power changes instead of wavelength shifts. Hence, the refractive index sensitivity has been established up to 2375.8 dB/RIU in the narrow RI range (1.333–1.341 RIU) and temperature sensitivities up to 1.1 dBm/°C in the range of 23–41 °C. The proposed sensor is dedicated to advanced chemical and biological sensor applications.

## 1. Introduction

Label-free monitoring of ambient refractive-index (RI) changes based on optical fiber sensing is a significant technology in biological [1], medical [2], and industrial [3] applications. Among the optical fiber configurations already proposed for RI sensing are surface plasmon interference [4], fiber Bragg gratings [5], long-period gratings (LPGs) [6], Mach-Zehnder interferometers [7], and Fabry-Perot interferometers [8]. These configurations have provided ultra-high sensitivity. However, they do not exclude cross-sensitivity derived from interaction with other physical parameters. In order to obtain a pure sensor response to the measured quantity, it is essential limiting the cross-sensitivities or controls two or more parameters at the same real time. The simultaneous measurement of several parameters is a well-established technique, and it can be achieved in optical devices by differential modulation [9], cascading [10], or multiplexing of two identical or different optical components [11,12]. Generally, such structures have been widely demonstrated for strain, temperature, and acoustic sensing [13], although multi-point RI measurements appear in few publications [14].

In recent years, special efforts in high-sensitivity RI monitoring have been directed to LPG-based sensors due to the strong relationship between the properties of the external medium and the spectral response of the LPG [15,16]. An LPG consists of a periodic modulation of the RI of the fiber core, as a result of which the fundamental core mode is coupled to higher-order cladding modes. This leads to the appearance of a series of resonant dips at discrete wavelengths. When external perturbations are imposed upon the LPG, the optical and expansion coefficients of the fiber material change. As a result, the resonant dips shift, and the amount of the spectral shift expresses the sensitivity of the sensor [17]. In the literature, the different modification of LPG technology can be monitored depending on application. A packaged long-period fiber grating (PLPFG) fabricated by the MEMS process and packaged with poly-dimentylsiloxane polimer materials has been proposed for the strain sensor [18,19]. An electroformed long-period fiber grating (ELPFG) with a periodic circle shaped polymer–metal structure fabricated by the lithography and electroforming processes has been proposed for magnetic field and temperature sensing [20,21]. A notched long-period fiber grating (NLPFG) using an inductively coupled plasma etching process has been also proposed for sensor applications, especially as a gas sensor [22,23].

In order to obtain an ultra-high RI sensitivity in an LPG-based sensor, the fundamental mode needs to couple to cladding modes of a specific higher order by using an appropriate grating period [24]. Such coupling leads to the simultaneous generation of a pair of resonance wavelengths caused by the particular dispersion characteristics of the modes [25]. In this approach, the cladding mode satisfies the phase-matching condition at two wavelengths, and such an LPG is called a dual-resonance long-period grating (DRLPG). It should be emphasized that the characteristic spectrum of a DRLPG works close to the dispersion turning point (DTP), which is exploited for the purpose of sensing in the ultra-sensitive zone [26], i.e., in the region where the sensitivity to any external perturbation is ultra-high. For this reason, the cross-sensitivity elimination or simultaneous measurement of more parameters is needed to filter out unexpected influences of the environment. Generally, an axial-strain cross-sensitivity can be resolved by stable construction of the LPG-based refractometer [27], but temperature influence is still a critical problem in sensor applications.

In this paper, the requirement to distinguish between sensor responses to RI and temperature have been met using a DRLPG combined with a fiber loop mirror (FLM) with a 3 dB coupler and a part of polarization-maintaining (PM) fiber [28]. Such an interferometer is the most suitable because two interacting beams propagate in the same fiber in opposite directions [29], and the general idea of proposed sensor platform has been already formulated in our paper [30]. Unfortunately, despite a number of the PM fiber advantages, there is a drawback: high thermal sensitivity. The solution of this problem is using the PM fiber also as a head for the temperature sensor. As long as the PM fiber is not stripped of the fiber coating, it is not susceptible to external RI variations. By virtue of its wavelength filtering properties, the FLM [31] enables monitoring of variations in external RI and temperature as an optical power ratio between the two interference dips [32]. Insertion of a polarization controller (PC) at a specific location in the interferometric structure provides an accurate adjustment of the fringe pattern with the two notches of the DRLPG. In this way, a measurement of ambient RI with temperature control is achieved by monitoring the two dips from the DRLPG and two separate dips that relate exclusively to interference.

## 2. Principle and Experimental Setup

### 2.1. Principle

The differences in the thermo-optic and thermo-expansion coefficients between the fiber core and the cladding material determine changes in the phase-matching conditions of the DRLPG. At the same time, RI variation also changes the phase-matching conditions, so that temperature-induced crosstalk in the DRLPG-based sensor is inevitable. Hence, as set out in [33], the response of the DRLPG to the surrounding RI and the temperature is expressed as:

(1) $$\Delta P_{DRLPG}=K_{DRLPG,n}\Delta n+K_{DRLPG,T}\Delta T$$

where $\Delta P_{DRLPG}$ is the optical power ratio between two peaks; $K_{DRLPG,n}$ and $K_{DRLPG,T}$ are the RI and the temperature sensitivity coefficients for the given DRLPG; ΔT and Δn are the ambient temperature and RI changes of the DRLPG.

As described above, the FLM contains PM fiber, whose birefringence directly varies with fluctuations in the surrounding temperature. As a result, the interference pattern displays shifts proportional to the temperature changes and, consequently, the amplitude of the peaks also varies. Given that coated PM fiber is insensitive to the ambient RI, the correlation between the interference optical power ratio of the FLM ($\Delta P_{FLM}$) and the surrounding temperature changes (ΔT) can be simply expressed as

(2) $$\Delta P_{FLM}=K_{FLM,T}\Delta T$$

where $K_{FLM,T}$ is the temperature sensitivity coefficient of the FLM.

According to the standard matrix inversion method [34] and assuming that the DRLPG and the FLM are both exposed to the same environment (real situation for FLM), the matrix relation can be generated from Equations (1) and (2) as follows:

(3) $$\left[\begin{array}{c} \Delta n\\ \Delta T \end{array}\right]={\left[\begin{array}{cc} K_{DRLPG,n} & K_{DRLPG,T}\\ 0 & K_{FLM,T} \end{array}\right]}^{-1}\times \left[\begin{array}{c} \Delta P_{DRLPG}\\ \Delta P_{FLM} \end{array}\right]\;$$

Each sensitivity coefficient and the response function can be obtained from the fitting curves of the experimental data. Given that the tested optical components were attached in the same place and the same stress conditions were provided for all tests, it is reasonable to assume that the matrix is not affected by random errors.

### 2.2. Experimental Setup

**Figure 1:**
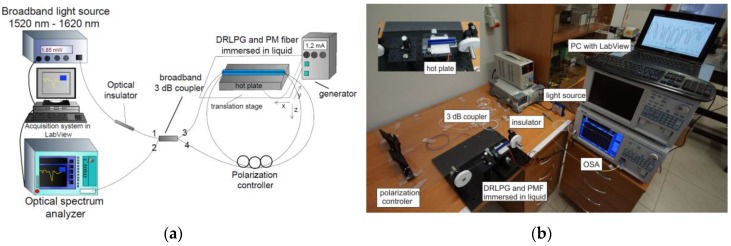
The schematic (**a**) and general photo (**b**) of experimental setup for the ambient refractive-index (RI) and temperature measurement sensor with emphasized the polarization controller (PC) position.

The proposed sensor (see Figure 1) consists of a broadband light source with a range of 1520–1620 nm, an optical insulator, a wideband single-mode 3 dB fiber-optic coupler, a 0.31 m length of PM fiber (HB 1500 bow-tie type, Fibercore, Southampton, UK), a manual fiber PC (FPC030 3-Paddle PC, Thorlabs, Newton, NJ, USA), a DRLPG, and an optical spectrum analyzer (OSA) (86142B, Agilent, Santa Clara, CA, USA) with a resolution of 0.1 nm [30]. The input light from port 1 passes through the 3 dB coupler and the optical insulator, which protects the light source from the back reflections. Here, light is split into two beams that counter-propagate from ports 3 and 4 and travel around the loop. The beams propagate simultaneously through the DRLPG and the PM fiber, so that their individual polarization varies and the beams interfere in output port 2. The OSA and light source were operated using a data acquisition system in the LabView environment, where the reference spectrum from the source was actually subtracted from the output signal in terms of limiting its influence on investigated features.

The DRLPG used in the experiment was fabricated with standard germanium-doped Corning SMF-28 fiber. For the LPG preparation, the chromium amplitude mask technique was used with a high-power KrF excimer laser (Pulse Master^®^-840, GSI Lumonics, Rugby, UK) emitting at 248 nm with 340 nJ the peak pulse energy [35]. The 4 cm–long bare fiber to be exposed to UV radiation was hydrogenated to make it photosensitive. The grating period was Λ = 217 µm, and the LPG was annealed at a temperature of 150 °C for 90 min in order to stabilize its optical properties. To obtain the dual-resonance of the transmission spectrum, the LPG was tuned by etching in 10% hydrofluoric acid (HF 10%), which slightly reduce the diameter of the fiber with rate estimated using the reference samples, to be 30 nm/min. The cladding of the LPG was etched until obtaining the dual-resonance. During that process, the resonant wavelength was shifted up to DTP [36].

When it comes to the proper investigation of the cladding mode order, in which the fundamental mode LP_0,1_ is coupled in DRLPG structure, a numerical simulations should be carried out. As one can find in [35,37,38], DRLPG fabricated in conditions described above coupled the fundamental mode with LP_0,9_ and LP_0,10_ modes when it is immersed, respectively, in water (*n* = 1.3333) and liquid with a higher refractive index (more than *n* = 1.3808). The collaboration with the above team of authors gives the verification of the experimental information about the most probably coupling modes.

**Figure 2:**
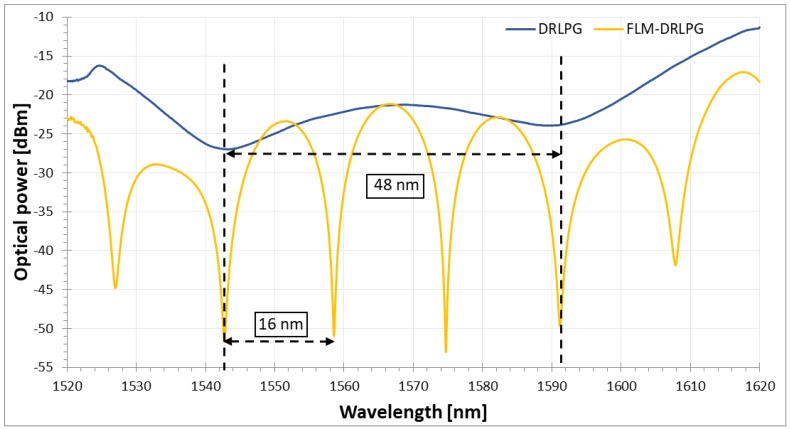
The transmission spectra of the dual-resonance long-period grating (DRLPG) (blue) and the DRLPG inside the fiber loop mirror (FLM) structure (yellow).

The proper wavelength characteristic is estimated by investigating PM fiber length and DRLPG wavelength influence [30,31]. The length of PM fiber was estimated in relation to the birefringence and the wavelength spacing between interference dips (16 nm) [39], which was matched to the spacing between DRLPG notches (48 nm) (Figure 2). The PC localization between the DRLPG and the PM fiber directly determines the peak amplitude and position. This implies that the PC can be used to control the behavior of the transmission spectrum and, hence, to tune the dips. For the type and length of PM fiber chosen in this test, it is possible to move the interference dips up to 9 nm and the amplitude about 11.25 dBm. For the final system adjustment, the two spectral peaks of the FLM were located in the middle of both notches of the DRLPG, as is shown in Figure 2.

In the presented experiment, the DRLPG served as the head of the ambient RI sensor, while the PM fiber functioned additional as the temperature sensor probe. The investigated liquid was dropped into the groove, and it did not leak due to the liquid viscosity. Both passive elements (PM fiber and DRLPG) were placed very close to each other in one groove in order to provide the same temperature conditions, although, for technical reasons, only one-fifth of the PM fiber was exposed to the temperature influence. The final temperature sensitivity estimate thus must be multiplied by five. To control temperature, the groove was attached to a ceramic hot plate, which was current controlled. The temperature was measured by a standard thermometer with a thermocouple. The DRLPG and the PM fiber were clamped on one side to the fiber holders and loaded with the same weight on the other side. This arrangement ensured the same axial stress on the DRLPG and the PM fiber during every test. The hot plate was mounted on the XYZ translation stage in order to enable a convenient and stable measurement procedure. All optical connections were made by fusion splicing with minimized connection losses. The measurement steps were as follows: aligning the DRLPG and the length of PM fiber in the central part of the groove; filling the groove with a liquid of known RI, setting the appropriate temperature, and registering changes in the transmission spectrum. The liquid was prepared by mixing water with glycerin in suitable proportions, and the RI value was then measured with an Abbe refractometer. Each test was performed after stabilization of the temperature. To avoid error, every measurement was preceded by thorough cleaning of the DRLPG with alcohol.

## 3. Results and Discussion

### 3.1. Temperature Response

**Figure 3:**
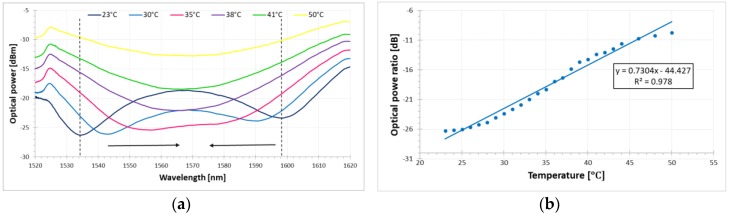
Transmission spectra of the DRLPG for (**a**) different temperatures in a liquid with RI value of *n* = 1.3333 and (**b**) optical power ratio between the two marked dashed line notches in figure (**a**) with a fitting linear function. The black arrows from (**a**) indicate the direction of the notches wavelength shifting.

The DRLPG was characterized in terms of ambient temperature variation. As can be seen in Figure 3a, the transmission spectrum significantly increases with temperature. A similar response is expected in the case of an ambient RI increase. Furthermore, both notches of the DRLPG are very flat, making it difficult to determine the minimum. Based on the changes in the signal’s spectrum, a ratio between the two notches (optical power ratio) and the measured linear response of the DRLPG to temperature fluctuations is calculated (see Figure 3b). From linear fitting, the maximum thermal sensitivity of the DRLPG can be evaluated at 0.7304 dB/°C with fitting coefficient equal to 0.978 for the temperature range of 23 °C up to 48 °C. As one can see in the border ranges of investigated temperature (below 25 °C and above 45 °C) flattening data is observed. It is probably caused by the properties of the liquid, as well as by experimental conditions. For the lower temperature range the liquid heat capacity limited hot plate proper operation in temperature similar to existing in laboratory (23 °C). Heating surrounding medium of the DRLPG to a higher temperature causes evaporating liquid from the vessel, and hence the boundary conditions of the DRLPG under tests were changed.

It is common knowledge that birefringence of a given PM fiber is strongly dependent on temperature. Thus, such a fiber in the FLM is very sensitive to the external temperature fluctuation [40]. In this case, the sensitivity of the temperature response is determined by both the birefringence and the length of the PM fiber employed. When the thermal response of a standard DRLPG is considered, the sensitivity is difficult to estimate due to the low extinction ratio of the notches, requiring advanced calculation procedures.

**Figure 4:**
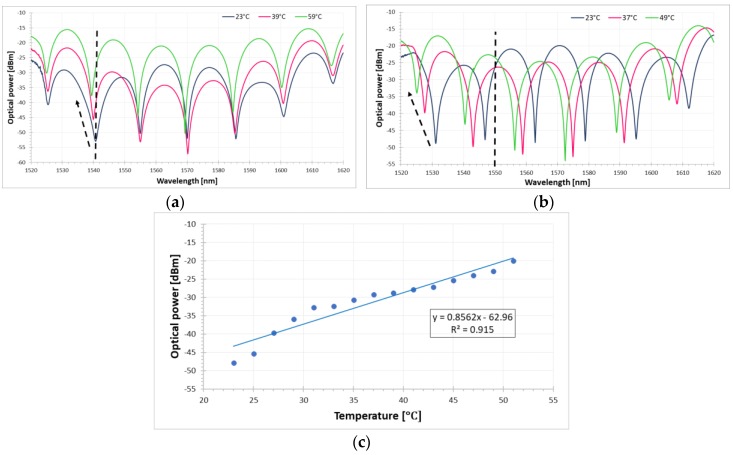
Transmission spectra of (**a**) DRLPG and (**b**) both PM and DRLPG for different temperatures in FLM-DRLPG structure. (**c**) Optical power of a single peak (λ = 1547 nm) versus external temperature with linear fit of the peak amplitude power changes.

Figure 4a shows the spectral response of the FLM-DRLPG structure where only the DRLPG was exposed to temperature changes. It can be seen that the peaks associated with both notches experiences a blue-shift, and at the same time, the amplitude decreases instead of covering the two DRLPG notches with interference dips when the temperature rises. In fact, where the dips do not overlap, the amplitude first increases and then decreases. This amplitude dip behavior can be explained by the thermal response of the DRLPG notches (around λ = 1542 nm and λ = 1590 nm), which move in opposite directions with rising temperatures. The spectral response is similar when the DRLPG is exposed to the ambient RI, making it crucial to distinguish between these two sensor signals. When both the DRLPG and the PM fiber in the FLM-DRLPG structure are simultaneously exposed to temperature influence (see Figure 4b), both the wavelength and the amplitude dips significantly change. In this case the temperature sensitivity is higher than for the DRLPG alone, a fact that can help avoid cross-sensitivity.

Figure 4c shows the FLM-DRLPG temperature response for a peak at λ = 1547 nm (see dashed line in Figure 4b) under a constant ambient RI (*n* = 1.3333). A linear fitting to the experimental data gives the amplitude sensitivity to the applied temperature and reaches about 0.8562 dBm/°C with worse form previously reported fitting coefficient equal to 0.915. The high scattering of measured points probably is related to the fact that sensitivity of the PM fiber to temperature change overlaps the spacing between interference patterns. The thermal sensitivity of the FLM-DRLPG structure is higher than it is for the pure DRLPG structure discussed above.

### 3.2. Ambient RI Response

**Figure 5:**
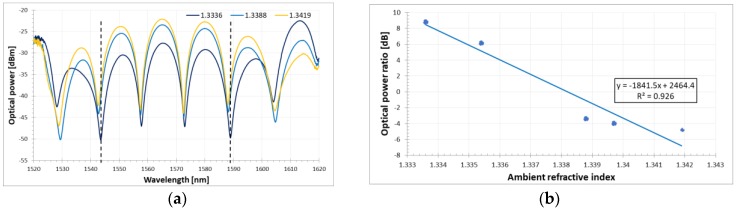
(**a**) Output transmission spectra of FLM-DRLPG structure for different ambient RI values at room temperature T = 23 °C and (**b**) sensor response for optical power ratio between the two peaks marked with dashed lines in (**a**).

The ambient RI response was measured in a temperature-controlled environment under variations of about 0.1 °C. A similar optical configuration was either proposed in terms of ambient RI measurement maximization [30]. However, the temperature influence has been completely omitted. The effect on the spectral response of the FLM-DRLPG structure induced by ambient RI variation is shown in Figure 5a. Despite the fact that the dips move slightly toward shorter wavelengths, the amplitude decreases with increasing RI of the liquid. For this reason, to estimate the ambient RI response of the setup, the ratio between the optical power of the two dips is monitored. This approach is justified because of the high extinction ratio of the existing interference dips in the FLM signal. The difference between the interference dips associated with the DRLPG notches is calculated, and the relationship between amplitude difference and ambient RI is also obtained. Through linear fitting (see Figure 5b), the sensitivity to the liquid RI variation is estimated to be 1841.5 dB/RIU with acceptable fitting coefficient equal to 0.926. In comparison with Chu el al. in [41], the present study obtained one order of magnitude higher ambient RI sensitivity expressed as the optical power ratio of the peaks.

It is worth noting that the performance of the proposed platform is affected by connection losses between the various optical components, by the sensitivity range of the OSA and by the bandwidth of the light source. In the experiment reported here, a low-power light source was used due to the short length of the sensor, and thus, the optical power of the presented transmission spectra is low. Special applications of the proposed sensor for remote measurement would require a higher-power optical source.

Since the coated PM fiber is completely insensitive to the ambient RI fluctuation, no result from this measurement is presented. Therefore, the ambient RI sensitivity of the FLM is assumed to be zero. From the results obtained and using the definitions from Equation (1), a sensitivity matrix can be established as follows:

(4) $$\left[\begin{array}{c} \Delta n\\ \Delta T \end{array}\right]={\left[\begin{array}{cc} 1841.5\;(\mathrm{dB}/\mathrm{RIU}) & 0.7304\;(\mathrm{dB}/{}^{\circ}C)\\ 0 & 0.8562\;(\mathrm{dBm}/{}^{\circ}C) \end{array}\right]}^{-1}\times \left[\begin{array}{c} \Delta P_{DRLPG}\\ \Delta P_{FLM} \end{array}\right]$$

In fact, both the DRLPG and the PM fiber are very sensitive to the temperature changes, so that two elements of the matrix, which correspond to the temperature sensitivity coefficient, have been obtained separately. This means that neither DRLPG nor PM fiber temperature sensitivity have influence to estimate temperature coefficient of sensitivity of this both elements. Since PM fiber is very susceptible to temperature disturbances, the thermal response was observed as the optical amplitude of the peak at λ = 1542 nm. For this reason, the thermal coefficient that stands for PM fiber temperature sensitivity is expressed as optical power in dBm/°C.

The resolution of measured parameters is conditioned by the reproducibility of the data and the amplitude resolution of the OSA, which was set as 0.02 dB. In this way, with sensitivity matrix, the RI and temperature of an unknown sample can be simultaneously measured.

### 3.3. Simultaneous Ambient RI and Temperature Measurement

**Figure 6:**
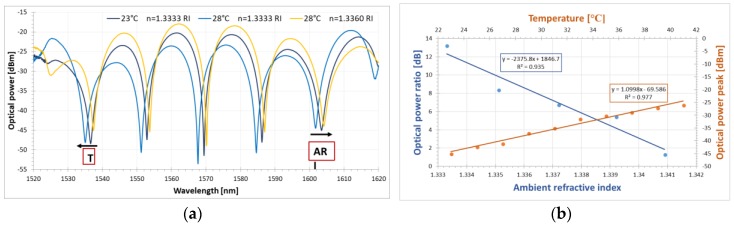
(**a**) Transmission spectra of FLM-DRLPG structure for ambient RI and temperature changes and (**b**) dual-parameter sensor response with linear fitting, where blue line stands for ambient RI response and orange line stands for ambient temperature response.

When both the ambient RI and the temperature change simultaneously, the transmission spectra are affected by both parameters. The measured response of the proposed sensor under these conditions is shown in Figure 6. It can be seen that when the temperature change is applied, the interference dips shift toward shorter wavelengths. On the other hand, when the ambient RI of the liquid is increased, the interference dips move toward longer wavelengths (see Figure 6a). It is noticeable that optical power for the dips varies as well, thus one can monitor the sensor response as optical power for dip change or as the optical power ratio between two interference dips. However, taking into account the ambient RI behavior of the transmission spectra of the DRLPG, one can monitor the response of the sensor as the optical power ratio of the two interference dips (λ = 1542 nm and λ = 1590 nm), corresponding to the two notches of the DRLPG. The sensor response for a chosen wavelength is presented in Figure 6b. In this case, the maximum ambient RI sensitivity of 2375.8 dB/RIU and temperature sensitivity of 1.1 dBm/°C with respective fitting coefficient equal to 0.935 and 0.977 is obtained. The relatively high scattering of the measuring points of the dual-parameter sensor may be attributed to the fact that the response is measured as an optical power for the dip change instead of a wavelength shift and the interference and resonance peaks should overlap in the entire RI and temperature measuring range. In this case, as shown in Figure 5a (wavelength between 1530 and 1540 nm), the optical power for the dip varies. To reduce scattering, in the future, a DRLPG with greater distance between the two LPG notches will be used. Of course, since sensitivity depends on the length or the birefringence of the PM fiber, or both, a longer fiber or one with higher birefringence is required to obtain greater thermal sensitivity.

The difference in the ambient RI sensitivities evident in the experimental data from Figure 5b and Figure 6b can be explained by the fact that the adjustment of the interference pattern may have been slightly different in the two cases, although the order of magnitude is still preserved. It is also possible that the PM fiber was subjected to incidental stress during the tests due to the length of the fiber, although each measurement was registered after stabilization time was allowed. To avoid any potential fluctuation of transmission spectra, a shorter PM fiber with higher birefringence could be used to maintain high thermal sensitivity of the sensor.

## 4. Conclusions

In conclusion, a refractive index sensor with temperature monitoring based on a DRLPG embedded inside an FLM is proposed in this paper. Cross-sensitivity can be avoided because the sensor exhibits opposite responses to variations in the ambient RI and temperature. The experimental results indicate that the transmission spectrum of the FLM-DRLPG structure can be considered as a superposition of the interference pattern of the FLM on the notches in the spectral response of the DRLPG. Temperature dependence of the DRLPG and PM fiber vary greatly. The PM fiber is more sensitive than DRLPG to the temperature fluctuation, as can be seen by comparing interference dips shifting from Figure 4a,b, respectively. Despite the fact that both the PM fiber and DRLPG suffered for temperature influence, in the presented platform, the DRLPG stands for an ambient RI detection, while the PM fiber serves as a temperature sensor and a band-pass filter for optical power ratio monitoring. PC-based tuning of the interference pattern by selection of an appropriate length of PM fiber ensures high ambient RI sensitivity expressed in the optical power ratio. Sensitivities of 2375.8 dB/RIU for the ambient RI measurement and almost 1.1 dBm/°C for temperature monitoring were obtained. These translate into 4.21 × 10^−4^ RIU/dB and 0.91 °C/dBm, respectively. However, the thermal sensitivity is mainly dependent on the length and birefringence of the PM fiber. For an amplitude stability of 0.02 dB of the OSA used, the minimum temperature and RI changes are, respectively, δT = 1.8 × 10^−2^ °C and δ*n* = 8.62 × 10^−6^ RIU. In terms of the ambient RI change from 1.333 up to 1.341, the sensor response range is almost 15 dB with the ratio of two optical power dips, whereas for temperatures from 23 °C to 41 °C the sensor response range is 20 dBm in terms of monitoring a single interference dip. When it comes to the wavelength shifting, the errors are estimated to be 0.4 nm and 0.3 nm for RI and temperature changing, respectively. The precision of amplitude dips is estimated to be 0.6 dBm for temperature and 0.4 dBm for RI measurement.

The proposed platform is easy to fabricate, and despite controlling two parameters, it provides high sensitivity of RI monitoring expressed in optical power ratio (in dB), which is required in great utility for advanced chemical and biological sensing applications.
